# Man or machine? Prospective comparison of the version 2018 EASL, LI-RADS criteria and a radiomics model to diagnose hepatocellular carcinoma

**DOI:** 10.1186/s40644-019-0266-9

**Published:** 2019-12-05

**Authors:** Hanyu Jiang, Xijiao Liu, Jie Chen, Yi Wei, Jeong Min Lee, Likun Cao, Yuanan Wu, Ting Duan, Xin Li, Ling Ma, Bin Song

**Affiliations:** 1 Department of Radiology, Sichuan University West China Hospital, No. 37 GUOXUE Alley, Chengdu, 610041 Sichuan China; 2 Department of Radiology & Institute of Radiation Medicine, Seoul National University Hospital, 101 Daehak-ro, Jongno-gu, Seoul, 03080 Republic of Korea; 3 Big data research center, University of Electronic Science and Technology of China, No. 2006 XIYUAN Avenue, West Hi-tech Zone, Chengdu, 610000 Sichuan China; 4 GE Healthcare, No.1 HUOTUO Road, Zhangjiang Hi-Tech Park, Pudong, Shanghai, 200000 China

**Keywords:** Carcinoma, Hepatocellular, Gadolinium ethoxybenzyl DTPA, Diagnosis, Machine learning, Guideline

## Abstract

**Background:**

The Liver Imaging Reporting and Data System (LI-RADS) and European Association for the Study of the Liver (EASL) criteria are widely used for diagnosing hepatocellular carcinoma (HCC). Radiomics allows further quantitative tumor heterogeneity profiling. This study aimed to compare the diagnostic accuracies of the version 2018 (v2018) EASL, LI-RADS criteria and radiomics models for HCC in high-risk patients.

**Methods:**

Ethical approval by the institutional review board and informed consent were obtained for this study. From July 2015 to September 2018, consecutive high-risk patients were enrolled in our tertiary care hospital and underwent gadoxetic acid-enhanced magnetic resonance (MR) imaging and subsequent hepatic surgery. We constructed a multi-sequence-based three-dimensional whole-tumor radiomics signature by least absolute shrinkage and selection operator model and multivariate logistic regression analysis. The diagnostic accuracies of the radiomics signature was validated in an independent cohort and compared with the EASL and LI-RADS criteria reviewed by two independent radiologists.

**Results:**

Two hundred twenty-nine pathologically confirmed nodules (173 HCCs, mean size: 5.74 ± 3.17 cm) in 211 patients were included. Among them, 201 patients (95%) were infected with hepatitis B virus (HBV). The sensitivity and specificity were 73 and 71% for the radiomics signature, 91 and 71% for the EASL criteria, and 86 and 82% for the LI-RADS criteria, respectively. The areas under the receiver operating characteristic curves (AUCs) of the radiomics signature (0.810), LI-RADS (0.841) and EASL criteria (0.811) were comparable.

**Conclusions:**

In HBV-predominant high-risk patients, the multi-sequence-based MR radiomics signature, v2018 EASL and LI-RADS criteria demonstrated comparable overall accuracies for HCC.

## Background

Hepatocellular carcinoma (HCC) is the fifth most common malignancy and the second leading cause of cancer-related death worldwide [1]. Currently, all major clinical guidelines [2–4] recommend the noninvasive diagnosis of HCC based on characteristic imaging findings on computed tomography, magnetic resonance (MR) imaging and/or contrast-enhanced ultrasound.

With the advent of novel imaging techniques, HCC diagnostic criteria have been continuously updated to incorporate several new imaging features on various modalities, among which the European Association for the Study of the Liver (EASL) criteria have been widely considered as a reliable scheme [2]. However, many of these criteria lack clear lexicons regarding modality-specific imaging features [2, 3]. Fortunately, the introduction of Liver Imaging Reporting and Data System (LI-RADS) offered the opportunity to standardize the interpretation, reporting and data collection of imaging results in patients at risk for HCC [5]. However, the assessment of several LI-RADS features can be subjective due to variations in radiologists’ experience and familiarity with the system [6, 7]. In addition, LI-RADS is developed and modified based predominantly on Western data [2, 4], but the demand for validation of the system in Asian cohort remains vital.

Radiomics, which allows quantitative tumor behavior and heterogeneity profiling by extracting high-throughput data with advanced image processing techniques [8], may be a possible approach to improve the accuracy and reproducibility of HCC diagnosis. Previous studies have demonstrated the potential of radiomics in the diagnosis of focal liver lesions [9] and several other solid tumors [10–12]. However, evidence regarding the comparison between the accuracies of radiomics models and existing HCC diagnostic criteria remains limited, and few studies have optimized the radiomics model with the multidisciplinary approach.

Thus, the aim of this prospective single-center study was to develop a diagnostic radiomics model for HCC and to compare its accuracy with the version 2018 (v2018) of the LI-RADS [5] and European Association for the Study of the Liver (EASL) criteria [2] in high-risk patients with surgical histopathologic examination as the reference standard. We also explored the diagnostic benefit of the refined radiomics-clinical model incorporating both radiomics features and predictive clinical markers.

## Methods

### Study cohort

Ethical approval by the institutional review board and informed consent from all patients were obtained for this prospective study before the start of patient enrollment. From July 2015 to September 2018, we enrolled consecutive adult patients with hepatitis B virus infection and/or cirrhosis to undergo gadoxetic acid (Gd-EOB-DTPA)-enhanced MR imaging from our tertiary care hospital. The exclusion criteria were patients i) with Child-Pugh class C disease; ii) with any previous antitumoral treatment (e.g. locoregional, surgical, systematic etc.); iii) with any contraindication of Gd-EOB-DTPA-enhanced MR imaging; iv) with inadequate image quality (e.g. substantial to severe arterial phase motion artifact); v) who did not receive or were not eligible for liver resection or transplantation in our center; vi) with inconclusive histopathologic diagnosis.

**Fig. 1:**
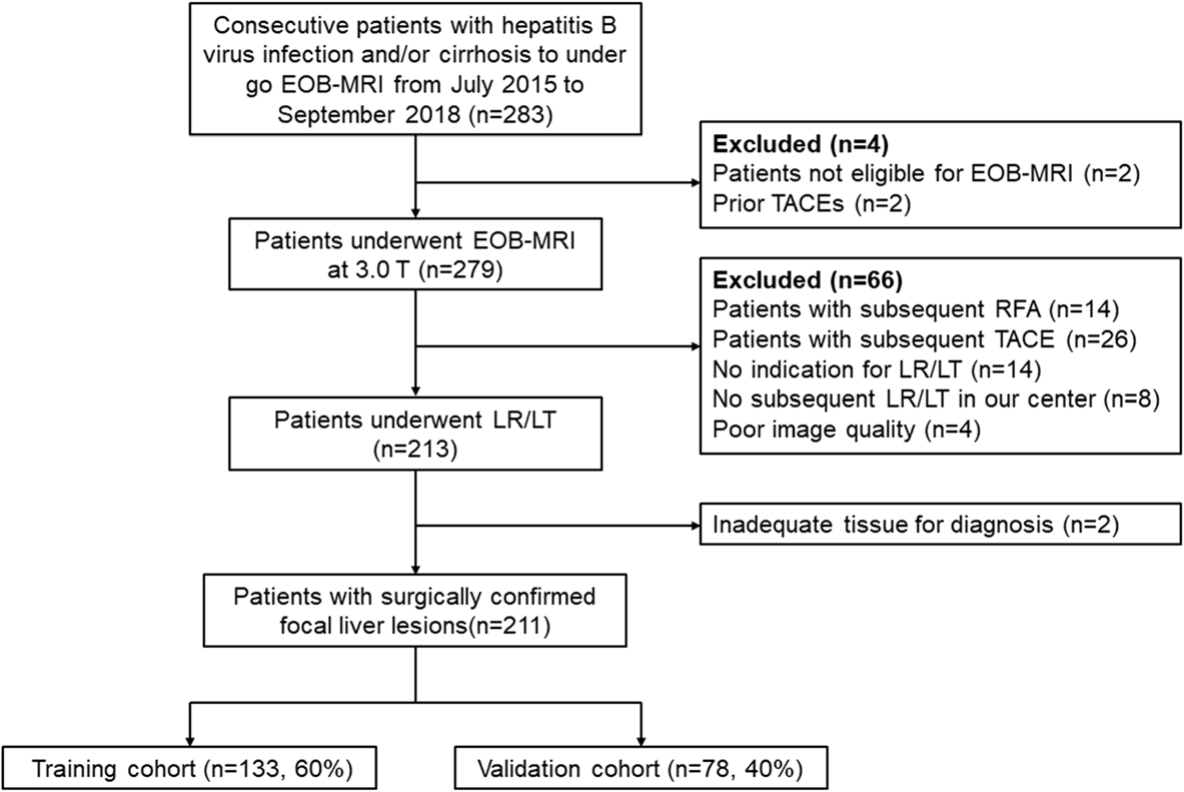
Study flow chart. US = ultrasound; CT = computed tomography; EOB-MRI = gadoxetic acid-enhanced magnetic resonance imaging; TACE = transarterial chemoembolization; RFA = radiofrequency ablation; LR = liver resection; LT = liver transplantation

A total of 283 patients were included during the study period, and 72 (26%) patients were excluded (Fig. 1). Therefore, the final study group included 211 patients (169 males, 80%).

### Imaging protocols

All MR examinations were performed on a MAGNETOM Skyra 3.0 T MR scanner (Siemens Healthcare, Erlangen, Germany). 0.025 mmol/kg of Gd-EOB-DTPA (Primovist®; Bayer Schering Pharma AG, Berlin, Germany) was injected at a rate of 2 ml/s. The detailed acquisition parameters were shown in the Additional file 1: Supplementary material and Table S1.

### Image analysis

#### Qualitative analysis

All MR imaging analyses were performed independently by two abdominal radiologists (with 10 years and 4 years of experience in liver imaging, respectively) who were blinded to the other imaging results, any clinical information and the final pathological diagnoses. Before start of the image analysis, both reviewers were given at least 2 months of intensive hands-on instructions in the practice of EASL v2018 and LI-RADS v2018 on Gd-EOB-DTPA-enhanced MR imaging.

Observations were diagnosed as HCC if they displayed a combination of arterial phase hyperenhancement and washout on portal venous phase exclusively by the EASL v2018 criteria [2]. Using all major, ancillary and LR-M features, each observation was assigned to an LR category according to the LI-RADS v2018 criteria by navigating the diagnostic algorithm in a stepwise fashion [5]. LR-4 V, LR-5 V or LR-MV was defined as LR-TIV contiguous with LR-4, LR-5 or LR-M lesions, respectively. All patient images were provided to the reviewers in random sequences, and both reviewers were asked to gap for at least 1 month between evaluating according to LI-RADS v2018 and evaluating according to EASL v2018 criteria. Disagreements regarding the LR categorization and HCC diagnosis were resolved by consensus with a senior abdominal radiologist with over 30 years of liver imaging experience.

#### Radiomics analysis

3D regions of interest were placed manually by delineating along the entire tumor margin on T2-weighted, T1-weighted in−/opposed-phase, unenhanced, arterial phase, portal venous phase, and hepatobiliary phase images to avoid major vessels and any marked necrotic areas with the 3D segmentation software ITK-SNAP [13] (version 3.6.0-RC1; *http://www.itk-snap.org*). The free-hand outlines were independently drawn by the two radiologists who conducted qualitative image analyses.

Radiomics analysis was performed with in-house texture analysis algorithms using the nonpublic scientific research 3D analysis software Analysis Kit (version v3.0.1. A, GE Healthcare, China). To standardize the imaging data of all MR images, the signal intensity is aligned to the same level by changing the formula of the original radiomics feature. In the processing of the pixel size, we pushed the wavelet transformation and calculated all features repeatedly. Using bin size as the variable point, one of the key processes in the standardization of feature extraction was feature discretion, which had a substantial impact on the value of the radiomics features. A total of 396 radiomics features from the categories of histogram, gray-level co-occurrence matrix, run-length matrix, gray-level size zone matrix, form factor and Haralick were extracted from each MR image.

#### Construction and validation of the radiomics models

All nodules were randomly divided into a training cohort (137 nodules [60%] in 133 patients) and a validation cohort (92 nodules [40%] in 78 patients) using repeated stratified splitting method to reduce the bias selection of a single validation dataset. In a multivariate analysis, the number of events should be no less than 10 times the number of included covariates [14]. Therefore, we applied the least absolute shrinkage and selection operator (LASSO) model [15] with 10-fold cross-validation to select radiomics features with the strongest diagnostic powers in the training data set. Radiomics features with an intraclass correlation coefficient over 0.80 between two reviewers were considered stable and entered into further radiomics model construction [16]. A radiomics score (Rad-score) of each MR sequence was calculated by a linear combination of the selected radiomics features weighted by the corresponding LASSO regression coefficients as:

$$ Rad- score={a}_1{X}_1+{a}_2{X}_2+\dots +{a}_n{X}_n+b $$

**Fig. 2:**
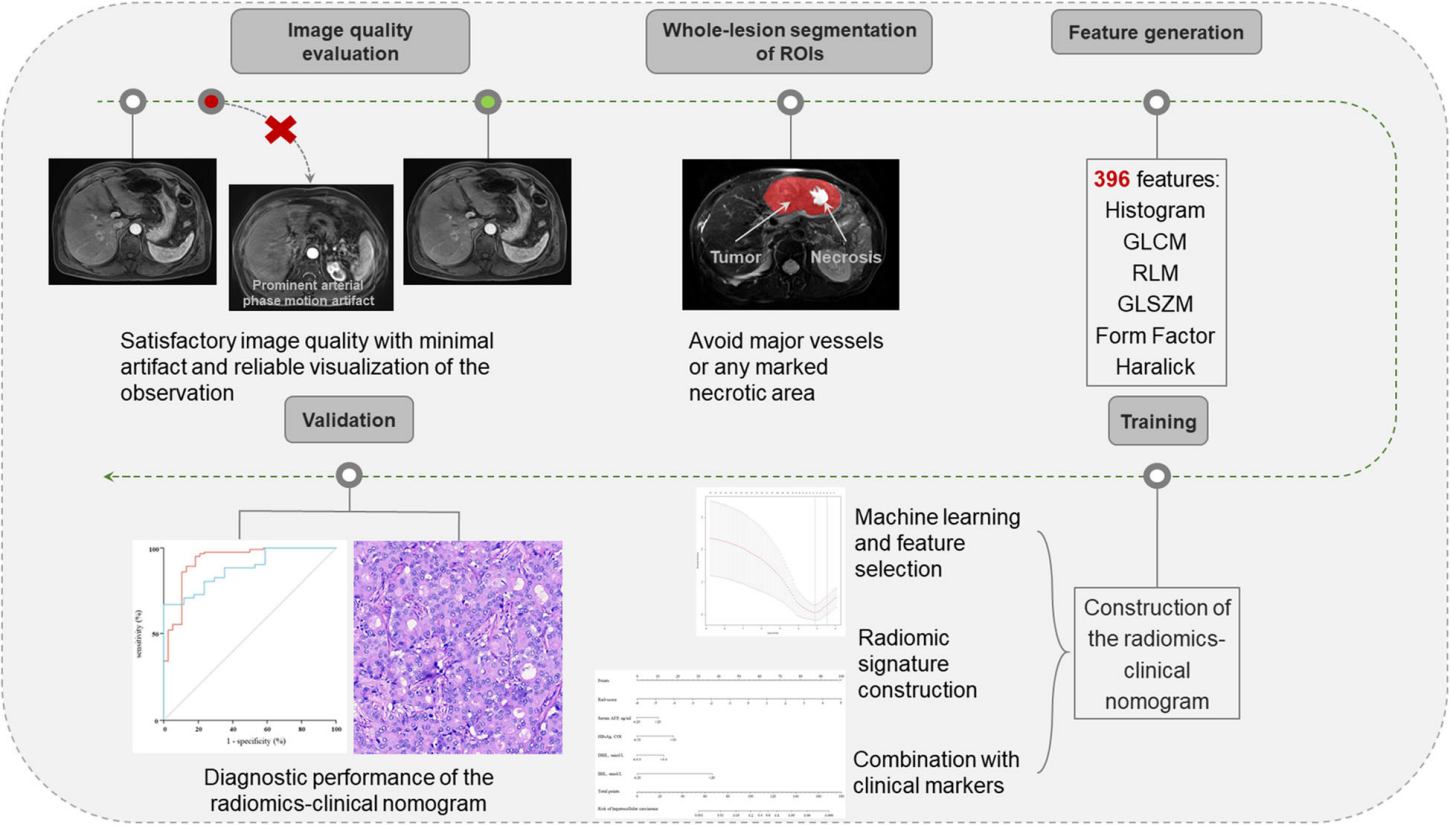
Workflow of construction and validation of the radiomics models. ROI = region of interest; GLCM = gray-level co-occurrence matrix; RLM = run-length matrix; GLSZM = gray-level size zone matrix

Where a_n_ is the LASSO regression coefficient of variable n, X_n_ is the value of the variable n determined from the input MR image and b is the intercept. A summarized Rad-score of all sequences was generated by a linear combination of the Rad-score of each sequence weighted by its logistic regression coefficient to construct the diagnostic radiomics signature. The radiomics signature was further integrated with clinical markers that were independently predictive for HCC diagnosis in the training cohort to formulate a radiomics-clinical nomogram with multivariate logistic regression analysis. The performances of the radiomics signature and radiomics-clinical nomogram were evaluated in the validation cohort (Fig. 2).

### Reference standard

Histopathologic examination of the resected or explanted liver was used as the reference standard for all lesions. Two experienced pathologists (with 8 years and over 20 years of experience in liver oncology, respectively), who were aware of the clinical data and imaging results for co-localization of the target lesions, independently performed gross and histologic analyses of all resected or explanted specimens. All disagreements were resolved by consensus. Histopathologic diagnoses of the hepatic lesions were established according to the World Health Organization classification [17].

### Statistical analyses

Differences were compared with Student’s test or the Mann-Whitney U test for continuous variables, and with χ^*2*^test or the Fisher’s exact test for categorical variables, where applicable. To identify clinical markers predictive of HCC, binary logistic regression analyses were used, and variables with a *p* value< 0.10 were further entered into the multivariate logistic analysis. Interrater reliability was evaluated with Cohen’s kappa coefficient (κ) for categorical variables. Agreement was considered poor (κ < 0.00), slight (κ: 0–0.2), fair (κ: 0.2–0.4), moderate (κ: 0.4–0.6), substantial (κ: 0.6–0.8) or excellent (κ: 0.8–1.0) [16] accordingly.

Per-lesion diagnostic performances were assessed by sensitivities, specificities, positive predictive values (PPVs), negative predictive values (NPVs) and receiver operating characteristic (ROC) analysis. Diagnostic measures were compared with the McNemar test or the method described by DeLong et al [18], where applicable. Comparisons of diagnostic accuracies between the EASL and LI-RADS criteria were conducted in the combined cohort comprising all patients, while all comparisons were made in the validation cohort between the radiomics signature and EASL or LI-RADS criteria.

All statistical analyses were performed with R software, version 3.3.1 (The R Foundation for Statistical Computing, Vienna, Austria). *P* values for multiple comparisons were adjusted by the Bonferroni method, and *p* < 0.05 was considered statistically significant.

## Results

### Patient characteristics

Demographic, clinical and biological information of the included patients is summarized in Table 1. A total of 173 nodules in 165 patients were proven as HCCs, 32 nodules in 30 patients as non-HCC malignancies (intrahepatic cholangiocarcinoma [ICCA]: *n* = 22; combined hepatocellular-cholangiocarcinoma [cHCC-CCA], *n* = 5; neuroendocrine tumor: *n* = 2; metastasis: *n* = 2; hemangiosarcoma: *n* = 1), and the remaining 24 nodules in 16 patients as non-HCC benign lesions (cavernous hemangioma: *n* = 6; angioleiomyolipoma: *n* = 6; focal nodular hyperplasia: *n* = 4; inflammatory pseudotumor: *n* = 4; dysplastic nodule: *n* = 3; hepatic adenoma: *n* = 1). Mean size of the included lesions was 5.43 cm (range: 1.0–14.9 cm). 34 (15%), 83 (36%) and 112 (49%) lesions were ≤ 2 cm, 2-5 cm and > 5 cm, respectively.

**Table 1:** Patient characteristics

Patient characteristics	HCC	non-HCC malignancies	non-HCC benign lesions
No. of patients	165	30	16
No. of lesions	173	32	24
Sex (male)	133 (80.6%)	24 (80.0%)	12 (75.0%)
Age (years, mean [range])	51.2 (26–83)	53.6 (39–77)	49.1 (30–60)
Size (cm, mean ± SD)	5.74 ± 3.17	5.64 ± 2.36	2.93 ± 2.43
Interval between MR imaging and surgery (d, mean ± SD)	2.73 ± 1.99	2.81 ± 2.25	4.62 ± 7.13
Underlying Diseases			
HBV-related cirrhosis	108	10	6
HBV carrier (not cirrhotic)	52	16	9
Cirrhosis of other causes	5	4	1
Child-Pugh Class			
A	164 (99.4%)	27 (90.0%)	16 (100.0%)
B	1 (0.6%)	3 (10.0%)	0
ALT (IU/L)			
>40	68 (41.2%)	6 (20.0%)	2 (12.5%)
AST (IU/L)			
>35	84 (50.9%)	7 (23.3%)	1 (6.3%)
TBIL (umol/L)			
>28.0	11 (6.7%)	4 (13.3%)	1 (6.3%)
IBIL (umol/L)			
>20.0	6 (3.6%)	5 (16.7%)	1 (6.3%)
ALB (g/L)			
<35	4 (2.4%)	2 (6.7%)	0
PT (s)			
>12.8	42 (25.5%)	4 (13.3%)	0
PLT (×10^9/L)			
<100	30 (18.2%)	3 (10.0%)	2 (12.5%)
HBsAg (COI)			
>10	105 (84.8%)	12 (40.0%)	9 (56.3%)
AFP (ng/ml)			
>20	105 (63.6%)	10 (33.3%)	1 (6.3%)
CEA (ng/ml)			
>3.4	29 (17.6%)	11 (36.7%)	1 (6.3%)
CA 19–9 (U/ml)			
>22	68 (41.2%)	15 (50.0%)	0

*Abbreviations*: *HCC* hepatocellular carcinoma, *SD* standard deviation, *MR* magnetic resonance, *CT* computed tomography, *HBV* hepatitis B virus, *NAFLD* non-alcoholic fatty liver disease, *ALT* alanine aminotransferase, *AST* aspartate aminotransferase, *TBIL* total bilirubin, *IBIL* indirect bilirubin, *ALB* albumin, *PT* prothrombin time, *PLT* platelet, *HBsAg* hepatitis B virus surface antigen, *AFP* alpha-fetoprotein, *CEA* carcinoembryonic antigen, *CA 19–9* carbohydrate antigen 19–9

Among the included patients, 201 (95%) were infected with HBV. No difference of the nodule type proportion (HCC, non-HCC malignancy and non-HCC benign lesion) or any demographic, clinical or biological characteristic was detected between the training and validation cohorts (*p* > 0.05 for all).

### Interrater agreement assessment

Table 2 summarizes the interrater reliability results of the EASL v2018 and different LI-RADS categories for all 229 nodules. Agreement was substantial between the two reviewers for each LI-RADS category (κ = 0.7437), the combination of LR-5/LR-5 V (κ = 0.6542), LR-4/LR-4 V/LR-5/LR-5 V (κ = 0.7109) and the EASL v2018 results (κ = 0.6809).

**Table 2:** Interrater reliability analysis of v2018 EASL and LI-RADS categories

LR categories for reviewer 1	LR category for reviewer 2										κ value	Agreement
	LR-1	LR-2	LR-3	LR-4	LR-4 V	LR-5	LR-5 V	LR-M	LR-MV	All		
Agreement on all LR categories												
LR-1	12	0	0	1	0	0	0	0	0	13	0.7437 (0.6644–0.8230)	Substantial
LR-2	0	2	0	0	0	0	0	1	0	3		
LR-3	0	0	0	0	0	0	0	0	0	0		
LR-4	1	1	0	6	0	6	0	0	0	14		
LR-4 V	0	0	0	0	0	2	0	1	0	3		
LR-5	0	0	0	7	0	118	2	1	0	128		
LR-5 V	0	0	0	0	0	2	27	1	1	31		
LR-M	0	0	1	1	0	7	3	13	3	28		
LR-MV	0	0	0	0	0	1	3	0	5	9		
All	13	3	1	15	0	136	35	17	9	229		
Agreement on LR-5/LR-5 V vs others												
Results for reviewer 1		Results for reviewer 2										
		LR-5/LR-5 V				others				All		
LR-5/LR-5 V		149				10				159	0.6542 (0.5453–0.7631)	Substantial
others		22				48				70		
All		171				58				229		
Agreement on LR-4/LR-4 V/LR-5/LR-5 V vs others												
Results for reviewer 1		Results for reviewer 2										
		LR-4/LR-4 V/LR-5/LR-5 V				others				All		
LR-4/LR-4 V/LR-5/LR-5 V		170				6				176	0.7109 (0.5985–0.8233)	Substantial
others		16				37				53		
All		186				43				229		
Agreement on EASL v2018												
Results for reviewer 1		Results for reviewer 2										
		0				1				All		
0		40				14				54	0.6809 (0.5674–0.7944)	Substantial
1		12				163				175		
All		52				177				229		

*Abbreviations*: *LI-RADS* Liver Imaging Reporting and Data System, *EASL* European Association for the Study of the Liver, *MRI* magnetic resonance imaging, *LR-4 V* LR-TIV in the presence of LR-4 lesions, *LR-5 V* LR-TIV in the presence of LR-5 lesions, *LR-MV* LR-TIV in the presence of LR-M lesions

Agreement was substantial to almost perfect for all LI-RADS major features and most ancillary and tiebreaking features (Additional file 2: Table S2). Agreement was not evaluated for nodule size or growth, which were provided to the reviewers.

### Construction and validation of the radiomics models

**Fig. 3:**
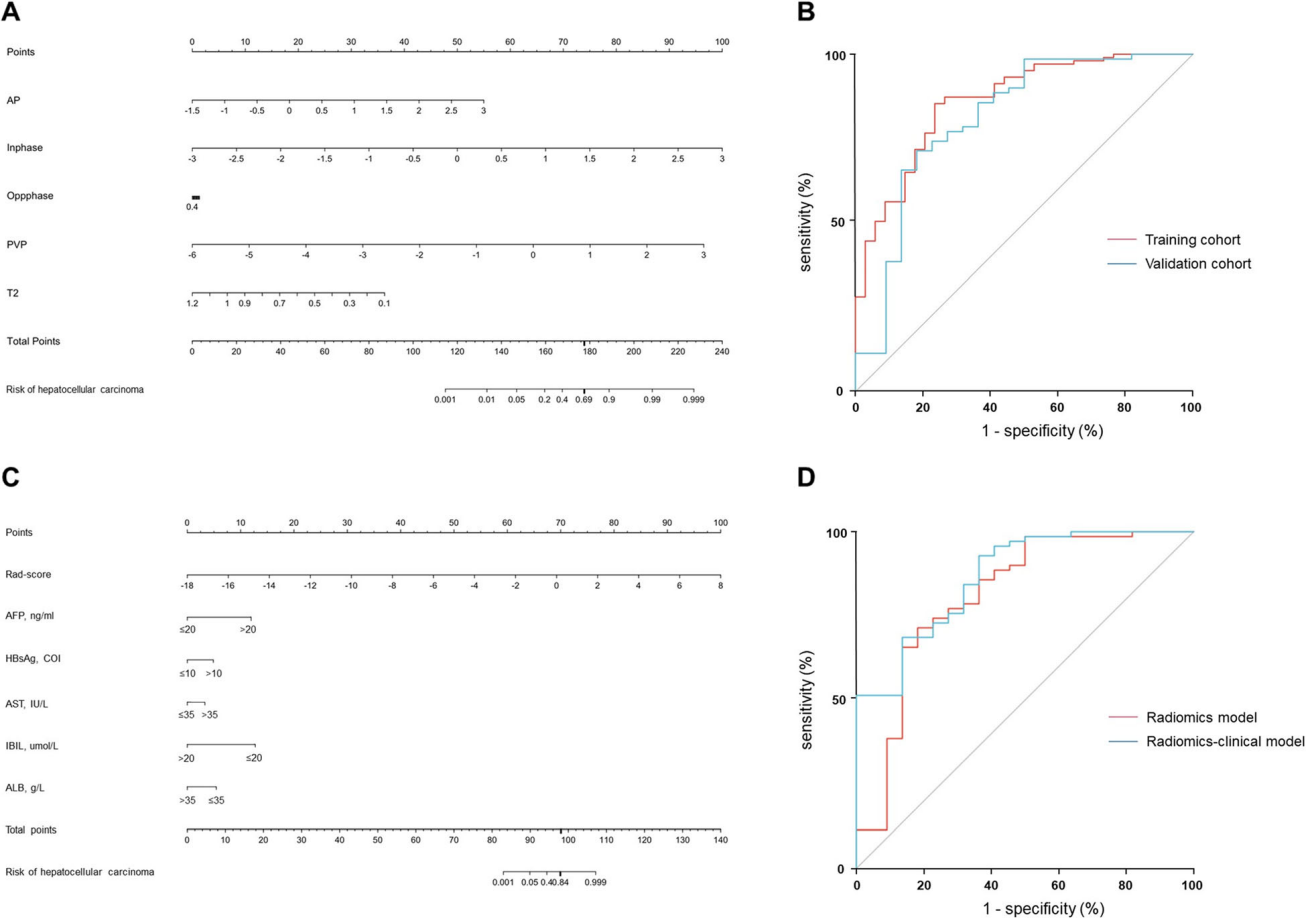
The radiomics models and their receiver operating (ROC) curves. The radiomics signature (**a**) and radiomics-clinical model (**c**) described in the form of nomograms to estimate the risk of a focal liver lesion to be HCC. Locate each variable on the corresponding axis, draw a line straight upward to the Points axis to determine the number of points, add the points from all the variables to get a total point, and draw a line straight down from the “Total Points” axis to the “Risk of hepatocellular carcinoma” axis to determine the HCC probability. **b** ROC curves of the radiomics signature in the training (red line) and validation cohorts (blue line). No difference (*p* = 0.521) (DeLong test) was detected between the area under the curve (AUCs) of the radiomics signature in the training cohort (0.861, 95%CI: 0.789–0.932) and in the validation model (0.810, 95%CI: 0.690–0.931). **d** ROC curves of the radiomics signature (red line) and radiomics-clinical model (blue line) in the validation cohort. No difference (*p* = 0.213) (DeLong test) was detected between the AUCs of the radiomics signature (0.810, 95%CI: 0.690–0.931) and the radiomics-clinical model (0.866, 95%CI: 0.782–0.951)

After LASSO regression analysis in the training data set, a total of 18 features with nonzero regression coefficients were extracted from T1-weighted in-phase, opposed-phase, arterial phase, portal venous phase images and T2-weighted images (Additional file 3: Table S3). After multivariate logistic regression analysis, the summarized Rad-score (Fig. 3a) revealing the radiomics information of all predictive sequences was generated as:

$$ {\displaystyle \begin{array}{l} Rad- score= in- phase\_ Rad- score\times 2.046+ opposed- phase\_ Rad- score\times 0.083+\\ {} arterial\ phase\_ Rad- score\times 1.500+ portal\ venous\ phase\_ Rad- score\times 1.316-T2-\\ {} weighted\ image\_ Rad- score\times 4.048+0.137\end{array}} $$

Serum AFP (*p*<0.001), HBsAg (*p* = 0.01), AST (*p* = 0.046), IBIL (*p*<0.001) and ALB (*p* = 0.049) were significantly predictive of HCC after multivariate logistic regression analysis in the training data set and were incorporated with the Rad-score to formulate a radiomics-clinical nomogram (Fig. 3c).

### Diagnostic accuracy of the radiomics models, EASL and LI-RADS criteria

Table 3 summarizes the diagnostic performances of the radiomics model, EASL and LI-RADS v2018 criteria by consensus.

**Table 3:** Diagnostic performances of the radiomics model, EASL and LI-RADS criteria

All lesions	EASL^a^	LI-RADS^a^			Radiomics model^b^	*p value*^‡^		
		L5/5V	L4/4V/5/5 V	*p value*^¶^		*EASL* vs *Rad*^d^	*EASL* vs *L5/5V*^¶^	*Rad* vs *L5/5V*^d^
All nodules, *n* = 229								
Sen	91% (95%CI: 0.85–0.95)	86% (95%CI: 0.80–0.91)	94% (95%CI: 0.89–0.97)	0.019	73% (95%CI: 0.61–0.83)	0.006	0.531	0.076
Spe	71% (95%CI: 0.58–0.83)	82% (95%CI: 0.70–0.91)	73% (95%CI: 0.60–0.84)	0.254	77% (95%CI: 0.55–0.92)	1.000	0.528	1.000
PPV	91% (95%CI: 0.87–0.94)	94% (95%CI: 0.89–0.86)	92% (95%CI: 0.87–0.94)	0.999	91% (95%CI: 0.8–0.97)	1.000	1.000	1.000
NPV	71% (95%CI: 0.60–0.80)	66% (95%CI: 0.56–0.74)	79% (95%CI: 0.67–0.87)	0.101	47% (95%CI: 0.3–0.65)	0.056	1.000	0.199
AUC	0.811 (95%CI: 0.754–0.859)	0.841 (95%CI: 0.787–0.886)	0.834 (95%CI: 0.780–0.880)	0.745	0.810 (95%CI: 0.690–0.931)	1.000	0.761	1.000
Cirrhotic liver, *n* = 136								
Sen	92% (95%CI: 0.85–0.96)	86% (95%CI: 0.78–0.92)	93% (95%CI: 0.86–0.97)	0.082	70% (95%CI: 0.55–0.83)	0.007	0.408	0.112
Spe	63% (95%CI: 0.41–0.81)	79% (95%CI: 0.58–0.93)	71% (95%CI: 0.49–0.87)	0.503	62% (95%CI: 0.32–0.86)	1.000	0.588	0.797
PPV	92% (95%CI: 0.87–0.95)	95% (95%CI: 0.90–0.98)	94% (95%CI: 0.89–0.97)	0.999	87% (95%CI: 0.72–0.96)	1.000	1.000	1.000
NPV	63% (95%CI: 0.45–0.77)	54% (95%CI: 0.42–0.66)	68% (95%CI: 0.51–0.81)	0.275	36% (95%CI: 0.17–0.59)	0.199	1.000	0.530
AUC	0.772 (95%CI: 0.693–0.840)	0.824 (95%CI: 0.750–0.884)	0.818 (95%CI: 0.743–0.879)	0.849	0.715 (95%CI: 0.524–0.906)	1.000	0.612	0.897
Non-cirrhotic liver, *n* = 93								
Sen	89% (95%CI: 0.78–0.95)	87% (95%CI: 0.76–0.94)	95% (95%CI: 0.86–0.99)	0.668	78% (95%CI: 0.56–0.93)	0.843	1.000	1.000
Spe	78% (95%CI: 0.60–0.91)	84% (95%CI: 0.67–0.95)	75% (95%CI: 0.57–0.89)	0.275	100% (95%CI: 0.66–1)	0.008	1.000	0.045
PPV	89% (95%CI: 0.80–0.94)	91% (95%CI: 0.82–0.96)	88% (95%CI: 0.80–0.93)	0.994	100% (95%CI: 0.81–1)	1.000	1.000	1.000
NPV	78% (95%CI: 0.63–0.88)	77% (95%CI: 0.63–0.87)	89% (95%CI: 0.72–0.96)	0.503	64% (95%CI: 0.35–0.87)	1.000	1.000	1.000
AUC	0.833 95%CI: 0.742–0.903)	0.856 (95%CI: 0.768–0.920)	0.850 (95%CI: 0.761–0.916)	0.852	0.923 (95%CI: 0.829–1)	0.631	1.000	0.981
≤2 cm, *n* = 34								
Sen	86% (95%CI: 0.64–0.97)	76% (95%CI: 0.53–0.92)	95% (95%CI: 0.76–1)	0.067	38% (95%CI: 0.09–0.76)	0.030	1.000	0.141
Spe	69% (95%CI: 0.39–0.91)	77% (95%CI: 0.46–0.95)	62% (95%CI: 0.32–0.86)	0.389	88% (95%CI: 0.47–1)	0.876	0.606	1.000
PPV	82% (95%CI: 0.66–0.91)	84% (95%CI: 0.66–0.94)	80% (95%CI: 0.67–0.89)	0.992	75% (95%CI: 0.19–0.99)	1.000	1.000	1.000
NPV	75% (95%CI: 0.50–0.90)	67% (95%CI: 0.47–0.82)	89% (95%CI: 0.53–0.98)	0.166	58% (95%CI: 0.28–0.85)	1.000	1.000	1.000
AUC	0.775 (95%CI: 0.599–0.900)	0.766 (95%CI: 0.589–0.893)	0.784 (95%CI: 0.610–0.906)	0.788	0.859 (95%CI: 0.658–1)	1.000	1.000	1.000
>2 cm, ≤5 cm, *n* = 83								
Sen	85% (95%CI: 0.74–0.93)	80% (95% CI: 0.68–0.89)	95% (95% CI: 0.86–0.99)	0.011	68% (95%CI: 0.45–0.86)	0.354	1.000	0.829
Spe	82% (95%CI: 0.60–0.95)	91% (95% CI: 0.71–0.99)	82% (95% CI: 0.60–0.95)	0.375	82% (95%CI: 0.48–0.98)	1.000	1.000	1.000
PPV	93% (95%CI: 0.84–0.97)	96% (95%CI: 0.87–0.99)	94% (95% CI: 0.86–0.97)	0.997	88% (95%CI: 0.64–0.99)	1.000	1.000	1.000
NPV	67% (95%CI: 0.51–0.79)	63% (95%CI: 0.50–0.74)	86% (95%CI: 0.66–0.95)	0.043	56% (95%CI: 0.30–0.80)	1.0001	1.000	1.000
AUC	0.835 (95%CI: 0.738–0.908)	0.856 (95%CI: 0.762–0.924)	0.885 (95%CI: 0.796–0.944)	0.466	0.806 (95%CI: 0.617–0.994)	1.000	1.000	1.000
>5 cm, *n* = 112								
Sen	96% (95%CI: 0.89–0.99)	92% (95% CI: 0.85–0.97)	92% (95% CI: 0.85–0.97)	1.000	83% (95%CI: 0.67–0.93)	0.063	1.000	0.514
Spe	62% (95%CI: 0.38–0.82)	76% (95% CI: 0.53–0.92)	71% (95% CI: 0.48–0.89)	0.725	67% (95%CI: 0.09–0.99)	1.000	0.933	1.000
PPV	92% (95%CI: 0.86–0.95)	94% (95%CI: 0.89–0.97)	93% (95%CI: 0.88–0.97)	0.999	93% (95%CI: 0.81–0.99)	1.000	1.000	1.000
NPV	76% (95%CI: 0.54–0.90)	70% (95%CI: 0.52–0.83)	68% (95%CI: 0.50–0.82)	0.920	22% (95%CI: 0.09–0.45)	1.000	1.000	1.000
AUC	0.788 (95%CI: 0.700–0.859)	0.842 (95%CI: 0.762–0.904)	0.819 (95%CI: 0.735–0.885)	0.317	0.746 (95%CI: 0.590–0.866)	0.303	0.915	0.627

*Abbreviations*: *EASL* European Association for the Study of the Liver, *LI-RADS* Liver Imaging Reporting and Data System, *L4* LR-4, *4 V* LR-TIV in the presence of LR-4 lesions, *L5* LR-5, *5 V* LR-TIV in the presence of LR-5 lesions, *Rad* radiomics model, *Sen* sensitivity, *Spe* specificity, *PPV* positive predictive value, *NPV* negative predictive value, *AUC* area under the receiver operating characteristic curve

^a^Diagnostic measures were evaluated in the combined cohort comprising all patients

^b^Diagnostic measures were evaluated in the validation cohort

^d^Comparisons were made in the validation cohort

‡*P* values were corrected with the Bonferroni method

¶Comparisons were made in the combined cohort comprising all patients

#### The radiomics models

The AUCs of the radiomics signature were 0.861 and 0.810 in the training and validation cohort, respectively (Fig. 3b). These measures were 0.982 and 0.866 for the radiomics-clinical nomogram in the training and validation cohort, respectively. In the validation cohort, the sensitivity, specificity, PPV and NPV of the radiomics signature and radiomics-clinical model were 73, 77, 91, 47 and 77%, 68, 89, 48%, respectively. No difference was detected between any paired diagnostic measure for the radiomics signature and radiomics-clinical model in the validation cohort (Fig. 3d) or for the radiomics signature in the training and validation cohorts (Fig. 3b).

#### EASL v2018

The sensitivity, specificity, PPV, NPV and AUC of the EASL v2018 criteria for all nodules were 91, 71, 91, 71% and 0.811, respectively. These measures were 92, 63, 92, 63%, and 0.772 for patients with cirrhosis, and 89, 78, 89, 78% and 0.833 for patients without cirrhosis. There’s no difference between any paired measures according to the status of underlying cirrhosis. When stratified by nodule sizes, the diagnostic accuracy was the highest in nodules > 2 cm but≤5 cm (AUC = 0.835, 95%CI: 0.738–0.908) and the lowest in nodules≤2 cm (AUC = 0.775, 95%CI: 0.599–0.900).

#### LI-RADS v2018

According to the LR categories in consensus, 0/13 (0%), 0/3 (0%), 13/17 (76.5%), 149/159 (93.7%) and 11/37 (29.7%) LR-1, LR-2, LR-4/LR-4 V, LR-5/LR-5 V and LR-M/LR-MV lesions were HCC, respectively. The per-lesion sensitivity, specificity, PPV, NPV and AUC for all nodules were 86, 82, 94, 66% and 0.841 by combination of LR-5/LR-5 V and 94, 73, 92, 79% and 0.834 by combination of LR-4/LR-4 V/LR-5/LR-5 V, respectively. The combination of LR-4/LR-4 V/LR-5/LR-5 V demonstrated a significantly higher sensitivity than LR-5/LR-5 V in all nodules (*p* = 0.02) and nodules between 2 and 5 cm (*p* = 0.01), without loss of specificity. However, the differences in AUCs between these two combinations were not significant (*p* = 0.32–0.85).

### Comparisons between the radiomics signature, the EASL and LI-RADS criteria

Diagnostic results by LR-5/LR-5 V were used to represent the LI-RADS v2018 performances. After *p* value adjustment for multiple comparisons, the v2018 EASL and LI-RADS criteria yielded comparable diagnostic accuracies for HCC irrespective of underlying cirrhosis or lesion size. In the validation cohort, the EASL v2018 demonstrated significantly higher sensitivity than the radiomics signature in all nodules (*p* = 0.01), cirrhotic livers (*p* = 0.01) and in nodules ≤2 cm (*p* = 0.03). The radiomics signature is more specific than the EASL (*p* = 0.01) and LI-RADS (*p* = 0.045) in non-cirrhotic livers. The AUCs of all three diagnostic models were comparable in the validation data set.

## Discussion

Both updated in 2018, the EASL and LI-RADS criteria are currently the most widely used diagnostic criteria for HCC. However, concerns have been raised for both criteria regarding their applicability in Asian cohort and with hepatobiliary-specific contrast agents. Advances in radiomics have led to improved tumor-heterogeneity quantification and may assist in liver lesion characterization [9]. In this prospective study, we found that the multi-sequence-based MR radiomics signature, the LI-RADS v2018 and the EASL v2018 demonstrated comparable diagnostic accuracies for HCC in high-risk patients.

First, we constructed a multi-sequence-based MR radiomics signature in the training cohort and compared its diagnostic accuracy with EASL and LI-RADS criteria exclusively in the validation cohort to eliminate the effect of overfitting. We found that the AUCs of the radiomics signature were similar to EASL and LI-RADS criteria irrespective of lesion size and the presence of underlying cirrhosis. Notably, in non-cirrhotic patients, the radiomics signature demonstrated 100% specificity, which was significantly higher than both EASL (*p* = 0.008) and LI-RADS (*p* = 0.045) criteria, with an excellent AUC of 0.923. Since HBV chronic infection is currently the leading risk factor for HCC in Asian countries [3] and in this context many HCCs can develop without cirrhosis, the radiomics signature may play a pivotal role in increasing the diagnostic specificity and overall accuracy for these patients. However, the radiomics signature was less sensitive than EASL criteria, particularly in cirrhotic livers and for lesions≤2 cm, and these might have been explained by the fact that radiomics signatures constructed in small lesions could not usually provide sufficient biological information in a reliable fashion, as many such small lesions have not developed in the full spectrum [19].

Extracted from clinical radiologic images, radiomics features can indicate the gene expression profiles of HCC [20] and reveal key phenotypic characteristics including tumor growth and vascular invasion [21–23]. In our multi-sequence-based radiomics signature, most extracted imaging features belonged to the gray-level co-occurrence matrix (61%, 11/18) and run-length matrix (28%, 5/18) categories. Gray-level co-occurrence matrix parameters can depict tumor texture described by pixel spatial relationships [24]. Run-length matrix features enable evaluation of the complex 3D structures labelled with the same grey level values and have been reported to indicate HCC aggressiveness on Gd-EOB-DTPA-enhanced MR imaging [19]. However, the one-to-one correlations between numerous radiomics features and complex tumor biology processes are still unclear and need to be explored in further studies.

Interestingly, we found that the radiomics-clinical model incorporating predictive clinical markers showed no diagnostic benefit compared with the sole radiomics signature. This finding highlighted the central role of imaging examinations in HCC diagnostic workflow and indicated that clinical markers may provide limited information for liver lesion characterization in high-risk patients.

Afterwards, we compared the performances between EASL and LI-RADS criteria in the combined cohort comprising all patients. Both criteria demonstrated similar diagnostic accuracies irrespective of lesion size and the underlying cirrhosis status, which were in line with the study of Ronot et al [25]. However, despite that both EASL and LI-RADS were developed and modified in order to be nearly 100% specific, we reported relatively low specificities of both criteria. These results were not in accordance with previous studies [25–28], in which the specificities of previous EASL and LI-RADS criteria reached up to 87.6–98.6% [25, 26] and 83.6–100% [25–28], respectively.

**Fig. 4:**
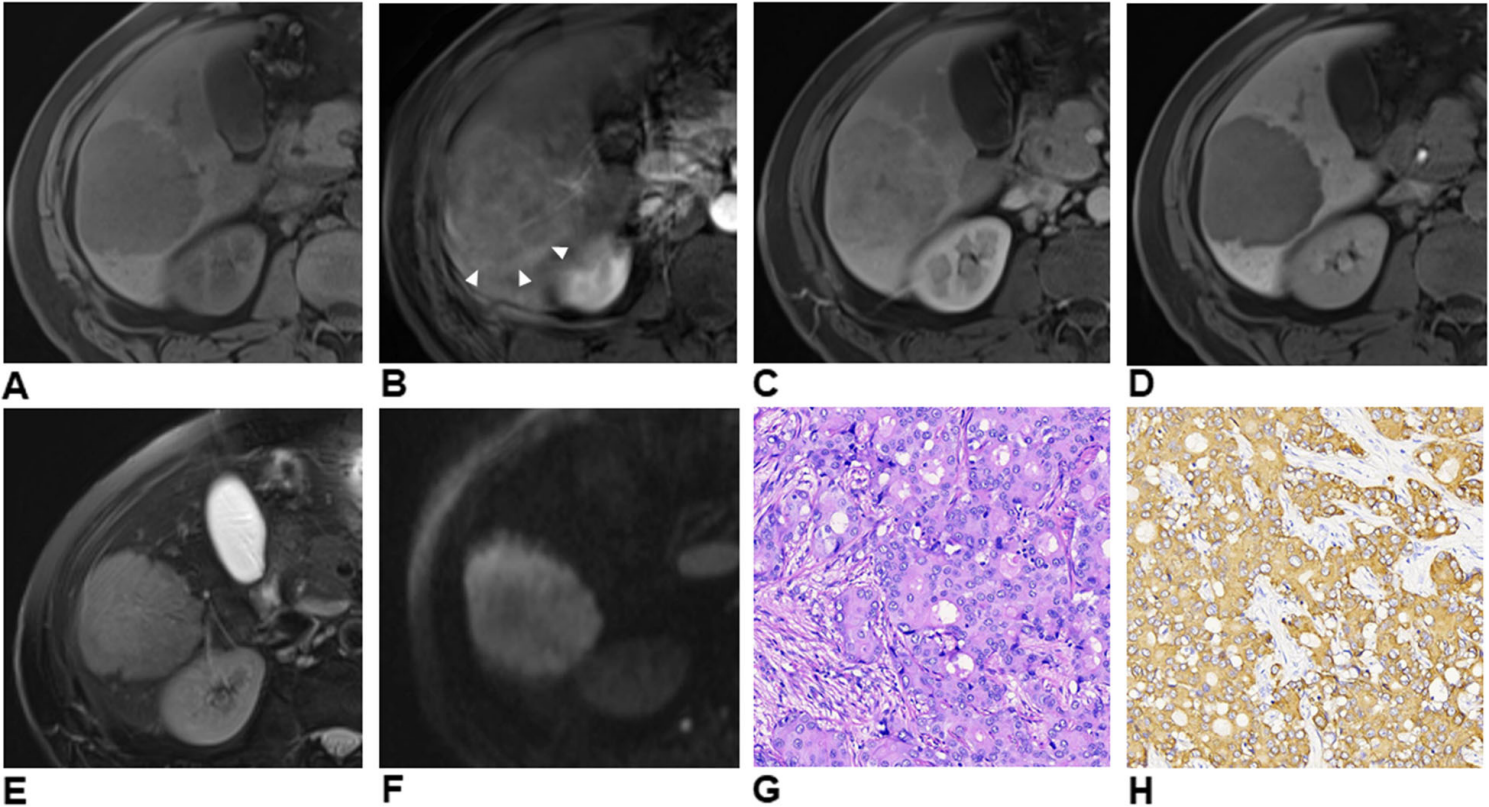
Gd-EOB-DTPA-enhanced MR images of a 47-year-old man with chronic HBV infection and pathologically proven cirrhosis. Images of un-enhanced phase (**a**) show a hypointense mass predominantly in segment VI. The mass demonstrates typical arterial phase (**b**) hyperenhancement (not rim), portal venous phase (**c**) washout and moderate T2 hyperintensity (**e**). No targetoid appearance is identified on hepatobiliary phase (**d**) or diffusion-weighted (**f**, b = 1200s/mm^2^) images. Note the peritumoral corona enhancement (**b**, white arrow heads) pattern in arterial phase due to venous drainage from the tumor. The mass was histopathologically proven to be intrahepatic cholangiocarcinoma with hematoxylin-eosin staining at 200 × magnification (**g**). Cytokeratin 19 is positive at 200 × magnification with immunohistochemical staining (**h**). The serum alpha-fetoprotein (4.91 ng/ml) and carcinoembryonic antigen 19–9 (17.44 U/ml) levels were within the normal range

Therefore, we explored origins of the restricted specificities on a per-lesion level. Among all false-positive cases, 9 (Fig. 4) were misclassified by both EASL and LI-RADS criteria (cHCC-CCA: *n* = 3; ICCA: *n* = 2; neuroendocrine tumor: *n* = 2; inflammatory pseudotumor: *n* = 1; angioleiomyolipoma: *n* = 1), 7 exclusively by EASL criteria (ICCA: *n* = 5; cHCC-CCA: *n* = 1; dysplastic nodule: *n* = 1) and 1 exclusively by LI-RADS criteria (ICCA). 85% (6/7) of the false-positive lesions misdiagnosed exclusively by EASL criteria presented the “targetoid appearance”, a target-like imaging morphology as a result of the highly cellular peripheral area surrounding the central fibrotic/ischemic stroma according to LI-RADS criteria [5]. This feature is highly indicative of ICCA, cHCC-CCA and other non-HCC malignancies. In our study, the “targetoid appearance” was significantly more common in non-HCC malignancies (75.0%) than in HCCs (7.5–9.8%) (both *p* < 0.001), as previously reported [7, 29]. Thus, a possible approach to improve the specificity of EASL criteria for HCC is to eliminate the effect of the “targetoid appearance” from the diagnostic algorithm.

However, neither EASL nor LI-RADS criteria demonstrated satisfactory specificities even after eliminating the effect of the “targetoid appearance”, particularly in differentiating between HCC and non-HCC malignancies in cirrhotic patients. One possible explanation was that 49% (112/229) of the included lesions were>5 cm. As larger lesions are more likely to demonstrate significant intratumoral heterogeneity and atypical imaging features, differential diagnosis of these tumors can be particularly challenging due to considerable clinical and imaging overlaps. By subgroup analysis, we reported the lowest specificities for both EASL and LI-RADS criteria in nodules>5 cm, which might have affected the overall diagnostic results substantially. Another likely explanation for the limited specificities was that 64% (134/211) of the included patients were cirrhotic, and small duct type ICCAs and cHCC-CCAs, can mimic HCCs in cirrhotic patients [30–32]. Similarly, Choi et al reported a relatively low specificity (87%) for LI-RADS v2017 in differentiating between HCC, ICCA and cHCC-CCA in HBV-predominant patients [32]. As both EASL and LI-RADS were developed in Western countries, where hepatitis C virus infection is the most important risk factor for HCC [2, 4], the diagnostic dilemma caused by these mimickers in chronic HBV patients may not be well addressed by either EASL or LI-RADS criteria.

In summary, the radiomics signature demonstrated comparable AUC for HCC with the v2018 EASL and LI-RADS but significantly higher specificity in non-cirrhotic patients, which may be clinically beneficial for patient with chronic HBV infection. However, the sensitivity of it was limited and the diagnostic results were difficult to interpret. In addition, radiomics results are prone to overfitting and the influence of imaging collection and modality variation [33, 34]. Thus, one of the key aspects of applying radiomics results in daily clinical practices is optimal acquisition and integration of curated data in a standardized and reproducible manner.

The EASL criterion is currently the most widely used diagnostic criteria for HCC. It is sensitive for small lesions, easy to apply and does not require the use of advanced imaging techniques. However, its accuracy might be restricted by relatively low specificity. LI-RADS empowers HCC probability assessment by integrating various imaging features with standardized interpretation and reporting. However, the diagnostic performances of LI-RADS were suboptimal in our HBV-predominant cohort. Apart from the geographical discrepancies of HCC between Western and Eastern cohorts, another possible explanation for the suboptimal performance of LI-RADS in this study might be the fact that LI-RADS was predominantly designed for MR using extracellular contrast agents instead of Gd-EOB-DTPA. Therefore, further tailoring of the system in Asian cohort using Gd-EOB-DTPA is necessary to optimize patient management. In addition, all LI-RADS ancillary features are weighted equally and optional, but some features (e.g. hepatobiliary phase hypointensity and restricted diffusion) may merit more emphasis or weighting [35]. Notably, combining LR-4 with LR-5 [26, 27] might be a possible approach to improve the sensitivity of LI-RADS in Eastern cohort.

This study has several limitations. First, the consecutive prospective cohort consisted of limited numbers of non-HCC and small HCC lesions. The small sample sizes of these specific categories of hepatic nodules might introduce significant selection bias to our diagnostic results. However, only patients with reliable pathological results were included, and many patients with small HCCs or non-HCC lesions were excluded because they were not candidates for surgery (e.g.*,* some non-HCC benign lesions), received alternative therapies (e.g.*,* ablation for small HCCs) or did not have conclusive histopathologic results. However, a different study design, such as using either histopathologic diagnosis or imaging follow-up as the reference standard might provide a larger number of these lesions. Second, we did not conduct multicenter external validation for the radiomics models due to dramatic variations in MR imaging protocols and surgical procedures across different centers. To overcome this limitation, we assessed the performance of the radiomics-clinical model in an independent validation cohort in our center. However, further prospective studies with multicenter large-scale external validation are warranted to assess the reproducibility and generalizability of the reported findings.

## Conclusions

The multi-sequence-based MR radiomics signature was significantly more specific in non-cirrhotic patients than v2018 EASL and LI-RADS criteria for HCC in HBV-predominant high-risk patients. However, the radiomics signature was less sensitive than v2018 EASL. The overall accuracies of these three diagnostic approaches were comparable.

## Supplementary information

**Additional file 1: **Detailed MR imaging acquisition protocols. All MR examinations were performed with an 18-channel body array coil. The MR sequences included: i) breath-hold fat-suppressed fast spin-echo T2-weighted imaging; ii) MR cholangiopancreatography heavily T2-weighted two-dimensional imaging, iii) in- and opposed-phase gradient-echo T1-weighted sequence, iv) diffusion-weighted sequence (b values: 0, 50, 500, 800, 1000, and 1200s/mm^2^), and v) a fat-suppressed three-dimensional (3D) gradient-echo T1 weighted sequence before and after intravenous injection of Gd-EOB-DTPA at the arterial phase ([AP] bolus triggering, 7 s after the signal intensity of the celiac trunk was the highest), portal venous phase ([PVP] 60–70s), transitional phase (3 min) and hepatobiliary phase ([HBP], 20 min). Injection of Gd-EOB-DTPA was immediately followed by a 30-ml saline flush through an antecubital venous catheter with a dual power injector. **Table S1.** MR Sequences and Parameters.

**Additional file 2: Table S2.** Frequencies of LI-RADS v2018 Features with Interrater Reliability Analysis.

**Additional file 3: Table S3.** Extracted Radiomics Features.

## Data Availability

The datasets of the current study would be available from the corresponding author on reasonable request.

## Abbreviations

AUC: Are under the receiver operating characteristic curve
cHCC-CCA: combined hepatocellular-cholangiocarcinoma
EASL: European Association for the Study of the Liver
Gd-EOB-DTPA: Gadoxetic acid
HCC: Hepatocellular carcinoma
ICCA: Intrahepatic cholangiocarcinoma
LASSO: Least absolute shrinkage and selection operator
LI-RADS: Liver Imaging Reporting and Data System
NPV: Negative predictive value
PPV: Positive predictive value

## References

[1] Akinyemiju T, Abera S, Ahmed M (2017). The burden of primary liver Cancer and underlying etiologies from 1990 to 2015 at the global, regional, and National Level: results from the global burden of disease study 2015. JAMA Oncol.

[2] Liver EAftSot (2018). EASL clinical practice guidelines: management of hepatocellular carcinoma. J Hepatol.

[3] Omata M, Cheng AL, Kokudo N (2017). Asia-Pacific clinical practice guidelines on the management of hepatocellular carcinoma: a 2017 update. Hepatol Int.

[4] Marrero JA, Kulik LM, Sirlin CB (2018). Diagnosis, staging, and Management of Hepatocellular Carcinoma: 2018 practice guidance by the American Association for the Study of Liver Diseases. Hepatology.

[5] Chernyak V, Fowler KJ, Kamaya A (2018). Liver imaging reporting and data system (LI-RADS) version 2018: imaging of hepatocellular carcinoma in at-risk patients. Radiology.

[6] Fraum TJ, Tsai R, Rohe E (2018). Differentiation of hepatocellular carcinoma from other hepatic malignancies in patients at risk: diagnostic performance of the liver imaging reporting and data system version 2014. Radiology.

[7] Kierans AS, Makkar J, Guniganti P (2019). Validation of liver imaging reporting and data system 2017 (LI-RADS) criteria for imaging diagnosis of hepatocellular carcinoma. J Magn Reson Imaging.

[8] Lambin P, Rios-Velazquez E, Leijenaar R (2012). Radiomics: extracting more information from medical images using advanced feature analysis. Eur J Cancer.

[9] Mougiakakou SG, Valavanis IK, Nikita A, Nikita KS (2007). Differential diagnosis of CT focal liver lesions using texture features, feature selection and ensemble driven classifiers. Artif Intell Med.

[10] Huang P, Park S, Yan R (2018). Added value of computer-aided CT image features for early lung Cancer diagnosis with small pulmonary nodules: a matched case-control study. Radiology.

[11] Kang D, Park JE, Kim YH (2018). Diffusion radiomics as a diagnostic model for atypical manifestation of primary central nervous system lymphoma: development and multicenter external validation. Neuro-Oncology.

[12] Bickelhaupt S, Jaeger PF, Laun FB (2018). Radiomics based on adapted diffusion kurtosis imaging helps to clarify Most mammographic findings suspicious for Cancer. Radiology.

[13] Yushkevich PA, Piven J, Hazlett HC (2006). User-guided 3D active contour segmentation of anatomical structures: significantly improved efficiency and reliability. Neuroimage.

[14] Harrell FE (2015). Regression modeling strategies with applications to linear models, logistic and ordinal regression, and survival analysis.

[15] Li Z, Sillanpaa MJ (2012). Overview of LASSO-related penalized regression methods for quantitative trait mapping and genomic selection. Theor Appl Genet.

[16] Fleiss JL, Cohen J (1973). The equivalence of weighted kappa and the intraclass correlation coefficient as measures of reliability. Educ Psychol Meas.

[17] Bosman FT, Carneiro F, Hruban RH, Theise ND (2010). WHO classification of Tumours of the digestive system.

[18] DeLong ER, DeLong DM, Clarke-Pearson DL (1988). Comparing the areas under 2 or more correlated receiver operating characteristic curves -a nonparametric approach. Biometrics.

[19] Zhou W, Zhang L, Wang K (2017). Malignancy characterization of hepatocellular carcinomas based on texture analysis of contrast-enhanced MR images. J Magn Reson Imaging.

[20] Haralick RM, Shanmugam K, Dinstein I (1973). Textural features for image classification. IEEE Trans Syst Man Cybern.

[21] Segal E, Sirlin CB, Ooi C (2007). Decoding global gene expression programs in liver cancer by noninvasive imaging. Nat Biotechnol.

[22] Wu M, Tan H, Gao F (2019). Predicting the grade of hepatocellular carcinoma based on non-contrast-enhanced MRI radiomics signature. Eur Radiol.

[23] Peng J, Zhang J, Zhang Q, Xu Y, Zhou J, Liu L (2018). A radiomics nomogram for preoperative prediction of microvascular invasion risk in hepatitis B virus-related hepatocellular carcinoma. Diagn Interv Radiol.

[24] Chen X, Cheung ST, So S (2002). Gene expression patterns in human liver cancers. Mol Biol Cell.

[25] Ronot M, Fouque O, Esvan M, Lebigot J, Aube C, Vilgrain V (2018). Comparison of the accuracy of AASLD and LI-RADS criteria for the non-invasive diagnosis of HCC smaller than 3cm. J Hepatol.

[26] Renzulli M, Biselli M, Brocchi S (2018). New hallmark of hepatocellular carcinoma, early hepatocellular carcinoma and high-grade dysplastic nodules on Gd-EOB-DTPA MRI in patients with cirrhosis: a new diagnostic algorithm. Gut.

[27] Min JH, Kim JM, Kim YK (2018). Prospective Intraindividual comparison of magnetic resonance imaging with Gadoxetic acid and extracellular contrast for diagnosis of hepatocellular carcinomas using the liver imaging reporting and data system. Hepatology.

[28] Basha MAA, AlAzzazy MZ, Ahmed AF (2018). Does a combined CT and MRI protocol enhance the diagnostic efficacy of LI-RADS in the categorization of hepatic observations? A prospective comparative study. Eur Radiol.

[29] Peporte AR, Sommer WH, Nikolaou K, Reiser MF, Zech CJ (2013). Imaging features of intrahepatic cholangiocarcinoma in Gd-EOB-DTPA-enhanced MRI. Eur J Radiol.

[30] Piscaglia F, Iavarone M, Galassi M (2015). Cholangiocarcinoma in cirrhosis: value of hepatocyte specific magnetic resonance imaging. Dig Dis.

[31] Joo I, Lee JM, Yoon JH (2018). Imaging diagnosis of intrahepatic and Perihilar Cholangiocarcinoma: recent advances and challenges. Radiology.

[32] Choi SH, Lee SS, Park SH (2019). LI-RADS classification and prognosis of primary liver cancers at Gadoxetic acid-enhanced MRI. Radiology.

[33] Lambin P, Leijenaar RTH, Deist TM (2017). Radiomics: the bridge between medical imaging and personalized medicine. Nat Rev Clin Oncol.

[34] Bi WL, Hosny A, Schabath MB (2019). Artificial intelligence in cancer imaging: clinical challenges and applications. CA Cancer J Clin.

[35] Kim YY, Choi JY, Sirlin CB, An C, Kim MJ (2019). Pitfalls and problems to be solved in the diagnostic CT/MRI liver imaging reporting and data system (LI-RADS). Eur Radiol.

