# Supplementary material for: Man or machine? Prospective comparison of the version 2018 EASL, LI-RADS criteria and a radiomics model to diagnose hepatocellular carcinoma
Source: Cancer Imaging. 2019 Dec 5;19:84. doi: 10.1186/s40644-019-0266-9 (PMC6896342; doi:10.1186/s40644-019-0266-9)
Supplement: Supplementary file 1 — Additional file 1: Detailed MR imaging acquisition protocols. All MR examinations were performed with an 18-channel body array coil. The MR sequences included: i) breath-hold fat-suppressed fast spin-echo T2-weighted imaging; ii) MR cholangiopancreatography heavily T2-weighted two-dimensional imaging, iii) in- and opposed-phase gradient-echo T1-weighted sequence, iv) diffusion-weighted sequence (b values: 0, 50, 500, 800, 1000, and 1200s/mm2), and v) a fat-suppressed three-dimensional (3D) gradient-echo T1 weighted sequence before and after intravenous injection of Gd-EOB-DTPA at the arterial phase ([AP] bolus triggering, 7 s after the signal intensity of the celiac trunk was the highest), portal venous phase ([PVP] 60–70s), transitional phase (3 min) and hepatobiliary phase ([HBP], 20 min). Injection of Gd-EOB-DTPA was immediately followed by a 30-ml saline flush through an antecubital venous catheter with a dual power injector. Table S1. MR Sequences and Parameters. [file 40644_2019_266_MOESM1_ESM.docx]

**Additional file 1: Detailed MR imaging acquisition protocols**

All MR examinations were performed with an 18-channel body array coil. The MR sequences included: i) breath-hold fat-suppressed fast spin-echo T2-weighted imaging; ii) MR cholangiopancreatography heavily T2-weighted two-dimensional imaging, iii) in- and opposed-phase gradient-echo T1-weighted sequence, iv) diffusion-weighted sequence (b values: 0, 50, 500, 800, 1000, and 1200s/mm^2^), and v) a fat-suppressed three-dimensional (3D) gradient-echo T1 weighted sequence before and after intravenous injection of Gd-EOB-DTPA at the arterial phase ([AP] bolus triggering, 7s after the signal intensity of the celiac trunk was the highest), portal venous phase ([PVP] 60-70s), transitional phase (3 min) and hepatobiliary phase ([HBP], 20min). Injection of Gd-EOB-DTPA was immediately followed by a 30-ml saline ﬂush through an antecubital venous catheter with a dual power injector.

**Table S1. MR Sequences and Parameters**

| Sequence | Fat suppression | TR  (ms) | TE  (ms) | Flip angle | Section thickness (mm) | Matrix size | Field of view (mm^2^) | Acquisition time (s) |
| --- | --- | --- | --- | --- | --- | --- | --- | --- |
| T2-weighted 2D TSE | Used | 2160 | 100 | 160° | 6 | 320×288 | 433×433 | 36 |
| Coronal T2-weighted HASTE | Used | 1000 | 96 | 129° | 3 | 320×320 | 400×400 | 25 |
| T1-weighted 3D GRE VIBE | Used | 3,95 | 1.92 | 9° | 2 | 352×256 | 400×296 | 14 |
| T1-weighted IP and OP imaging | Not used | 81 | 1.4 | 70° | 6 | 352×286 | 400×325 | 24 |
| DW single-shot spin-echo EPI | Used | 5600 | 68 | 90° | 6 | 100×76 | 380×289 | 233 |
| MRCP T2-weighted HASTE | Used | 4500 | 709 | 180° | 40 | 384×269 | 300×300 | 4 |

*Abbreviations: TR=repetition time; TE=echo time; 2D=two-dimensional; 3D=three-dimensional; TSE=turbo spin-echo; HASTE=half fuorier single-shot turk spine-echo; GRE=gradient recall echo; VIBE=volume interpolated breath-hold examination; IP=in-phase; OP=opposed-phase; DW=diffusion-weighted; EPI=echo planar imaging; MRCP=magnetic resonance cholangiopancreatography.*
