# Supplementary material for: Man or machine? Prospective comparison of the version 2018 EASL, LI-RADS criteria and a radiomics model to diagnose hepatocellular carcinoma
Source: Cancer Imaging. 2019 Dec 5;19:84. doi: 10.1186/s40644-019-0266-9 (PMC6896342; doi:10.1186/s40644-019-0266-9)
Supplement: Supplementary file 2 — Additional file 2: Table S2. Frequencies of LI-RADS v2018 Features with Interrater Reliability Analysis. [file 40644_2019_266_MOESM2_ESM.docx]

**Table S2: Frequencies of LI-RADS v2018 Features with Interrater Reliability Analysis**

| **Imaging feature** | **HCC** | **non-HCC malignancies** | **non-HCC benign lesions** | **κ value** | **Agreement** |
| --- | --- | --- | --- | --- | --- |
| **Major features** | | | | | |
| *APHE* | | | | | |
| *Any type* | | | | 0.8905 (0.7848-0.9962) | Excellent |
| R1 | 166/173 (96.0%) | 32/32 (100.0%) | 9/24 (37.5%) |  |  |
| R2 | 169/173 (97.7%) | 32/32 (100.0%) | 10/24 (41.7%) |  |  |
| *Nonrim* | | | | 0.8263 (0.7390-0.9136) | Excellent |
| R1 | 155/173 (89.6%) | 11/32 (34.4%) | 6/24 (25.0%) |  |  |
| R2 | 164/173 (94.8%) | 12/32 (37.5%) | 6/24 (25.0%) |  |  |
| *Rim* | | | | 0.7824 (0.6644-0.9003) | Substantial |
| R1 | 11/173 (6.4%) | 21/32 (65.6%) | 3/24 (12.5%) |  |  |
| R2 | 5/173 (2.9%) | 20/32 (62.5%) | 4/24 (16.7%) |  |  |
| *Washout* | | | | | |
| *Any type* | | | | 0.7553 (0.6512- 0.8594) | Substantial |
| R1 | 158/173 (91.3%) | 16/32 (50.0%) | 3/24 (12.5%) |  |  |
| R2 | 164/173 (94.8%) | 17/32 (53.1%) | 1/24 (4.2%) |  |  |
| *Nonperipheral* | | | | 0.8437 (0.7648-0.9227) | Excellent |
| R1 | 155/173 (89.6%) | 8/32 (25.0%) | 2/24 (8.3%) |  |  |
| R2 | 160/173 (92.5%) | 10/32 (31.3%) | 1/24 (4.2%) |  |  |
| *Peripheral* | | | | 0.6484 (0.4218-0.8750) | Substantial |
| R1 | 3/173 (1.7%) | 8/32 (25.0%) | 1/24 (4.2%) |  |  |
| R2 | 4/173 (2.3%) | 7/32 (21.9%) | 1/24 (4.2%) |  |  |
| *Enhancing “capsule”* | | | | 0.7321 (0.6413-0.8228) | Excellent |
| R1 | 131/173 (75.7%) | 7/32 (21.9%) | 7/24 (29.2%) |  |  |
| R2 | 118/173 (68.3%) | 14/32 (22.2%) | 6/24 (25.0%) |  |  |
| **LR-M features** | | | | | |
| *Targetoid mass* | | | | 0.8123 (0.7139-0.9106) | Excellent |
| R1 | 17/173 (9.8%) | 24/32 (75.0%) | 3/24 (12.5%) |  |  |
| R2 | 13/173 (7.5%) | 24/32 (75.0%) | 4/24 (16.7%) |  |  |
| *Infiltrative appearance* | | | | 0.8738 (0.7882-0.9594) | Excellent |
| R1 | 28/173 (16.2%) | 8/32 (25.0%) | 2/24 (8.3%) |  |  |
| R2 | 29/173 (16.8%) | 8/32 (25.0%) | 1/24 (4.2%) |  |  |
| *Necrosis or severe ischemia* | | | | 0.5371 (0.4278-0.6463) | Moderate |
| R1 | 94/173 (55.0%) | 14/32 (40.7%) | 4/24 (25.0%) |  |  |
| R2 | 97/173 (32.6%) | 13/32 (3.7%) | 4/24 (50.0%) |  |  |
| **Ancillary features** | | | | | |
| Favoring malignancy, not HCC in particular | | | | | |
| *Corona enhancement* | | | | 0.7367 (0.6477-0.8257) | Substantial |
| R1 | 79/173 (45.7%) | 17/32 (53.1%) | 1/24 (4.2%) |  |  |
| R2 | 68/173 (39.3.2%) | 18/32 (56.3%) | 0 |  |  |
| *Fat sparing in solid mass* | | | | 0.6892 (0.4985-0.8799) | Substantial |
| R1 | 17/173 (9.8%) | 3/32 (9.4%) | 0 |  |  |
| R2 | 11/173 (6.4%) | 3/32 (9.4%) | 0 |  |  |
| *Restricted diffusion* | | | | 0.9691 (0.9086-1.0000) | Excellent |
| R1 | 173/173 (100%) | 32/32 (100.0%) | 6/24 (87.5%) |  |  |
| R2 | 173/173 (100%) | 32/32 (100.0%) | 7/24 (75.0%) |  |  |
| *Mild-moderate T2 hyperintensity* | | | | 0.7691 (0.6242-0.9141) | Substantial |
| R1 | 172/173 (99.4%) | 27/32 (84.4%) | 7/24 (29.2%) |  |  |
| R2 | 172/173 (99.4%) | 30/32 (94.8%) | 7/24 (29.2%) |  |  |
| *Iron sparing in solid mass* | | | | … | … |
| R115 | 0 | 0 | 0 |  |  |
| R2 | 0 | 0 | 0 |  |  |
| *Transitional phase hypointensity* | | | | 0.5755 (0.3677-0.7833) | Moderate |
| R1 | 166/173 (96.0%) | 26/32 (81.3%) | 19/24 (79.2%) |  |  |
| R2 | 168/173 (97.1%) | 25/32 (78.1%) | 19/24 (79.2%) |  |  |
| *Hepatobiliary phase hypointensity* | | | | 0.9311 (0.7966-1.0000) | Excellent |
| R1 | 167/173 (97.7%) | 32/32 (100.0%) | 22/24 (75.0%) |  |  |
| R2 | 168/173 (96.9%) | 32/32 (100.0%) | 22/24 (75.0%) |  |  |
| Favoring HCC in particular | | | | | |
| *Nonenhancing “capsule”* | | | | 0.6029 (0.4487-0.7570) | Substantial |
| R1 | 26/173 (15.0%) | 2/32 (6.3%) | 3/24 (12.5%) |  |  |
| R2 | 24/173 (13.9%) | 1/32 (3.1%) | 5/24 (20.8%) |  |  |
| *Nodule-in-nodule architecture* | | | | 0.6427 (0.5441-0.7414) | Substantial |
| R1 | 103/173 (59.5%) | 4/32 (12.5%) | 2/24 (8.3%) |  |  |
| R2 | 110/173 (65.6%) | 6/32 (18.8%) | 2/24 (8.3%) |  |  |
| *Mosaic architecture* | | | | 0.5707 (0.4619-0.6796) | Moderate |
| R1 | 126/173 (72.9%) | 12/32 (33.3%) | 1/24 (4.1%) |  |  |
| R2 | 123/173 (75.2%) | 12/32 (33.3%) | 2/24 (8.3%) |  |  |
| *Fat in mass, more than adjacent liver* | | | | 0.5215 (0.4037-0.6393) | Moderate |
| R1 | 70/173 (40.5%) | 3/32 (9.4%) | 5/24 (20.8%) |  |  |
| R2 | 62/173 (35.8%) | 5/32 (15.6%) | 4/24 (16.7%) |  |  |
| *Blood products in mass* | | | | 0.6478 (0.5446-0.7510) | Substantial |
| R1 | 76/173 (37.2%) | 6/32 (25.9%) | 0 |  |  |
| R2 | 71/173 (41.1%) | 8/32 (18.5%) | 0 |  |  |
| Favoring benignity | | | | | |
| *Parallels blood pool enhancement* | | | | … | … |
| R1 | 0 | 0 | 0 |  |  |
| R2 | 0 | 0 | 0 |  |  |
| *Undistorted vessels* | | | | … | … |
| R1 | 0 | 0 | 0 |  |  |
| R2 | 0 | 0 | 0 |  |  |
| *Iron in mass, more than liver* | | | | 0.6647 (0.0467-1.0000) | Substantial |
| R1 | 2/173 (1.2%) | 0 | 0 |  |  |
| R2 | 1/173 (0.6%) | 0 | 0 |  |  |
| *Marked T2 hyperintensity* | | | | 0.8573 (0.7198-0.9948) | Substantial |
| R1 | 1/173 (0.6%) | 0 | 15/24 (62.5%) |  |  |
| R2 | 0 | 0 | 15/24 (62.5%) |  |  |
| *Hepatobiliary phase isointensity* | | | | 0.6601 (0.2993-1.0000) | Substantial |
| R1 | 3/173 (1.7%) | 0 | 1/24 (4.1%) |  |  |
| R2 | 4/174 (2.3%) | 0 | 1/24 (4.1%) |  |  |
| **Tiebreaking features** | | | | | |
| *Tumor in vein* | | | | 0.7749 (0.6697-0.8802) | Substantial |
| R1 | 38/173 (22.0%) | 6/32 (18.8%) | 0 |  |  |
| R2 | 33/173 (19.1%) | 12/32 (37.5%) | 0 |  |  |

*Abbreviations: HCC=hepatocellular carcinoma; LI-RADS=* *Liver Imaging Reporting and Data System; APHE=arterial phase hyperenhancement; R1=reviewer 1; R2=reviewer 2.*
