# Supplementary material for: Man or machine? Prospective comparison of the version 2018 EASL, LI-RADS criteria and a radiomics model to diagnose hepatocellular carcinoma
Source: Cancer Imaging. 2019 Dec 5;19:84. doi: 10.1186/s40644-019-0266-9 (PMC6896342; doi:10.1186/s40644-019-0266-9)
Supplement: Supplementary file 3 — Additional file 3: Table S3. Extracted Radiomics Features. [file 40644_2019_266_MOESM3_ESM.docx]

**Table S3. Extracted Radiomics Features**

| **Feature group** | **Histogram features** | **Run-length matrix (RLM) features** | **Grey level co-occurrence matrix (GLCM) features** | **Haralick features** |
| --- | --- | --- | --- | --- |
| **T1-weighted in-phase** | Kurtosis | Cluster Prominence | Energy, Inertia, Inverse Difference Moment, Sum Entropy | - |
| **T1-weighted opposed-phase** | - | - | Correlation | - |
| **T1-weighted arterial phase** | - | Long Run High Grey Level Emphasis | Cluster Shade, Inverse Difference Moment |  |
| **T1-weighted portal venous phase** | - | Cluster Prominence, Long Run Emphasis | Correlation, Energy, Entropy, Inverse Difference Moment | Haralick Correlation |
| **T2-weighted images** | - | Cluster Prominence | - | - |
